# Foot Cooling between Interval Bouts Enhances Repeated Lower Limb Power Performance: The Role of Delaying Fatigue

**DOI:** 10.5114/jhk/159623

**Published:** 2023-01-20

**Authors:** Chih-Min Wu, Jen-Yu Ho, Wen-Yi Wang, Zong-Yan Cai

**Affiliations:** 1 Department of Leisure and Sport Management, Cheng Shiu University, Kaohsiung, Taiwan.; 2 Department of Athletic Performance, National Taiwan Normal University, Taipei, Taiwan.; 3 Graduate Institute of Sports Pedagogy, University of Taipei, Taipei, Taiwan.; 4 Center for Physical and Health Education, Si Wan College, National Sun Yat-sen University, Kaohsiung, Taiwan.

**Keywords:** leg press, cold immersion, power output, arousal, electromyography

## Abstract

This study aimed to investigate whether interbout foot cooling (FC) may enhance repeated lower limb power performance and the corresponding physiological responses based on interset FC, which has been demonstrated to enhance leg-press performance. In a repeated-measures crossover design, ten active men (aged 21.5 ± 1.5 years, exercising >3 times per week) performed four bouts of 10-s cycle ergometer sprints with interbout FC at 10°C water for 2.5 min or non-cooling (NC) with a 5-day interval. The results indicated that FC elicited higher total work (27.57 ± 5.66 kJ vs. 26.55 ± 5.76 kJ) and arousal scores than NC (p < 0.05). Furthermore, under the NC condition, participants decreased mean power (p < 0.05) with no alteration of vastus lateralis (VL) electromyography (EMG) activities after the second bout; whereas under the FC condition, participants maintained steady mean power accompanied by increased VL EMG activities in the last two bouts (p < 0.05). Jointly, participants had higher mean power ([3^rd^ = 10.14 ± 1.15 vs. 9.37 ± 1.30; 4^th^ = 9.79 ± 1.22 vs. 9.23 ± 1.27] W/kg) and VL EMG activities in the last two bouts under the FC than NC condition (p < 0.05). However, perceived exertion and the heart rate were comparable between the two conditions (p > 0.05). In conclusion, interbout FC elicited a higher arousal level and repeated lower limb power performance, which could be explained by delaying peripheral fatigue via increasing excitatory drive and recruiting additional motor units to compensate for fatigue-related responses and power decrements.

## Introduction

Athletes often perform repeated bursts of maximal effort and sprints with a brief recovery period in exercise scenarios (Natera et al., 2020). Therefore, strategies that help athletes to exert superior lower limb power and accelerate recovery between bouts to attenuate performance decrements are vital for the training process as well as to succeed in many sports.

Recent studies report that foot cooling (FC) improves lower limb strength performance in moderate indoor ambient temperature (Cai et al., 2021; Wu et al., 2021). In resistance-trained athletes, during four sets of 85%–90% one- repetition maximum (1 RM) leg-press exercise pyramid workouts, interset FC at 10°C for 2.5 min exhibits higher repetitions leading to exhaustion per set, higher total exercise volume without increasing the degree of rating of perceived exertion (RPE), and greater vastus lateralis (VL) muscle activation than non-cooling (NC) conditions (Cai et al., 2021). In a more recent study, intermittent FC at 10°C for 2.5 min before attempting maximal strength tests resulted in increased 1 RM leg-press strength (7.1%) and higher arousal levels, and recruited more motor units of the agonist muscles during the 1 RM test (Wu et al., 2021). FC, a type of peripheral cooling, refers to the cooling the extremities (palms and feet) that are distal to the exercising muscles (Cai et al., 2021; Caruso et al., 2015; Kwon et al., 2010) or cooling the joints (ankles and knees) adjacent to the exercising muscles (Hopkins et al., 2002; Palmieri-Smith et al., 2007; Pietrosimone and Ingersoll, 2009) rather than cooling the muscles themselves. Peripheral cooling may induce cooling afferent stimuli to the neuromuscular region (Hopkins et al., 2002; Palmieri-Smith et al., 2007) while preventing a decline in muscle temperature that affects muscle contraction velocity (Cross et al., 1996; Ruiz et al., 1993). Collectively, this strategy has boosted strength performance in remote muscle groups from the cooling area, e.g., FC increased leg-press performance (Cai et al., 2021; Wu et al., 2021), and palm cooling increased subsequent sets of bench press performance (Kwon et al., 2010, 2015).

Although the precise mechanisms underlying the improvement in multiple sets of strength performance by peripheral cooling are unclear, these mechanisms appear to involve inducing central nervous system arousal (Hopkins et al., 2002; Kwon et al., 2010; Palmieri-Smith et al., 2007; Pietrosimone and Ingersoll, 2009), facilitating motor neuron pool excitability (Cai et al., 2021; Hopkins et al., 2002; Kwon et al., 2010), and delaying the onset of fatigue (Caruso et al., 2015; Kwon et al., 2010, 2015). Strength gains may potentiate power performance (Chelly et al., 2009; Seitz et al., 2014). Intermittent FC aids leg-press performance either at high intensity or at 1 RM conditions; this raises the possibility that the further increased strength by FC could enhance repeated bursts of lower limb power performance

Therefore, this study aimed to investigate whether the application of acute FC between bouts has an ergogenic effect on repeated power performance. We used a 10-s sprint-cycling modality workout including four bouts. This approach was used because 4–6 bouts of the 10-s sprint-cycling protocol have previously been used as an interval training workout and performing them regularly has been found to increase power output (Hazell et al., 2010). If an interbout FC is beneficial for repeated power performance, it can be used as an ergogenic strategy for increasing power output and eliciting better training outcomes. Moreover, physiological and perceptual responses during the workout were measured as secondary outcomes. We hypothesised that FC would increase the power output and arousal level, amplify agonist muscle activation, and reduce the heart rate (HR) and RPE response during the workout.

## Methods

### Participants

Ten physically active men (mean ± standard deviation [SD]; age: 21.5 ± 1.5 years; body height: 178.4 ± 4.8 cm; body mass: 68.4 ± 6.4 kg) participated in this study. The eligibility criteria were as follows: physically active men aged ≥20 years who had been performing regular exercise (>3 times/week) for at least 6 months and were classified as healthy individuals with no major medical history. The exclusion criteria were as follows: a history of lower limb injury in the previous 6 months, fear of cooling and taking ergogenic supplements or medicine that could affect exercise performance. Participants were instructed to maintain their usual diet during the study, but refrain from strenuous exercise, caffeine, and alcohol 24 h before each trial and to avoid having a pre-workout meal 1–3 h before each condition (Caruso et al., 2015). In addition, they had to cease their regular exercises during the study period.

The sample size was calculated using G*Power software, version 3 (Franz Faul, Christian-Albrechts-Universität Kiel, Germany), based on the data from a previous study that investigated the effects of FC on repeated high-intensity leg-press working volume (Cai et al., 2021). To detect statistically significant differences in 1-RM leg-press strength with 80% power, we used an alpha level of 5% and an effect size (ES) of 1.259 following a previous study (Cai et al., 2021). At least six participants were required.

The recruited participants were fully informed about the experimental procedure, potential risks and benefits of the study. They signed informed consent forms before their participation. The study was conducted according to the guidelines of the Declaration of Helsinki and approved by the National Cheng Kung University Human Research Ethics Committee (NCKU HREC-E-110-440-2).

### Design and Procedures

The experiments were conducted over 3 days, including one day of familiarisation followed by two days of repeated sprint-cycling. A randomised, counterbalanced, within-group research design was used to evaluate the research question and test our hypotheses. Each participant served as his own control. Participants performed four bouts of 10-s cycle ergometer sprints at room temperature under NC and interbout FC conditions, within a 5-day interval.

At first, participants were familiarized with the study procedures; that day also included collecting individual anthropometric data as well as adjusting the seat height, the handlebar height and position, and toe straps attached to the mechanically braked cycle ergometer (Ergomedics 874, Monark, Sweden). During the repeated sprint-cycling workout days, Ag/AgCl circular bipolar surface electrodes (2 cm interelectrode distance) were attached to the VL of the dominant leg to collect EMG signals during each bout of sprint cycling. Moreover, participants wore a Garmin Forerunner 935 Fitness Watch (Garmin International, Inc., Kansas, USA) for HR measurement. After the placement, participants rested for 10–15 min. Subsequently, they performed a standard warm-up of 5-min cycling at 60–80 rpm with no resistance.

**Figure 1:**
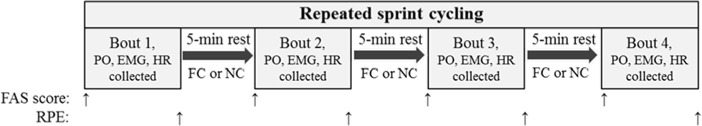
Experimental design: procedures for four bouts of 10 s sprint cycling under the FC and NC conditions, including a schedule of variables collected. PO = power output; EMG = electromyography; HR = heart rate; FAS = felt arousal scale; RPE = rating of perceived exertion; FC = foot cooling; NC = non-cooling.

Following the warm-up, participants performed four bouts of 10-s all-out effort cycling, separated by 5 min of recovery with or without interbout FC. Before each bout, their arousal levels were assessed using the felt arousal scale (FAS). Immediately at the end of each bout, they were asked to rate their RPE for the entire 10-s duration (Figure 1). All experiments were performed at the same time of day for each participant in a normothermic room, which was maintained at a temperature of 24°C–26°C and humidity of 40%–60%. All experimental days were conducted under the strict and direct supervision of at least two experienced researchers familiar with sprint cycling.

### Measures

#### FC Intervention

The cooling method with cold-water immersion has been applied in many studies (Cai et al., 2021; Caminita et al., 2020; Wu et al., 2021). In the present study, participants immersed their feet up to the distal end of the fibula (lateral malleolus) in buckets filled with water controlled at 10°C ± 1°C, as previously described (Cai et al., 2021; Wu et al., 2021). The water temperature throughout the FC condition was monitored, and the target temperature was maintained by adding crushed ice when needed (Cai et al., 2021; Wu et al., 2021). Benefits on leg-press performance have been observed after the application of this temperature during foot immersion (Cai et al., 2021; Wu et al., 2021). Under the FC condition, the water cooled the feet for 2.5 min between bouts of sprint cycling (Figure 1), whereas under the NC condition, the feet were placed in buckets, but without cold water.

#### Repeated Power Exercise Workout

All the participants performed a standard warm-up of 5-min cycling at 60–80 rpm with no resistance. After resting for 5 min, they performed four bouts of 10-s sprint-cycling against a resistance equal to 0.075 kg per kg of body mass. Participants then began to pedal as fast as possible against the inertia of the initial resistance in the first 2 s. At this stage, data on the power output and EMG values were excluded from the analysis. These values were selected for statistical analysis only in the subsequent 10-s sprint-cycling during each bout (Hazell et al., 2010). Participants were verbally encouraged during each cycling bout. Upon completion of four bouts of the sprint-cycling workout, they performed a 2-min cool down of cycling at a slow pace (<60 rpm) against no resistance. Values for the total work volume, mean power, and peak power were recorded and computed during each bout. In this study, mean power was defined as the average power output recorded during each 10-s cycling bout, whereas peak power (W•kg^-1^) was defined as the highest power output obtained at any moment during each 10-s cycling bout expressed in relative values (Zajac et al., 1999).

#### FAS

The FAS is used in the literature as a measure of perceived arousal (Svebak and Murgatroyd, 1985). Immediately before beginning each bout, participants had to indicate their perceived arousal using a poster containing the six-point FAS (Svebak and Murgatroyd, 1985). Verbal anchors were identified with high scores described by states (e.g., excitement) and with low scores described by states (e.g., relaxation).

#### EMG Signal Analysis

Surface electrodes were attached to the belly of the VL muscles of the dominant leg, whereas the ground electrode was placed over the epicondyle of the tibia after shaving, abrading and cleaning the participant’s skin with alcohol to minimise impedance. Electrode placement was marked for the following assessments. Participants removed these marks until the two sprint cycling trails were completed. EMG signals were collected and processed using the Noraxon Telemyo DTS EMG system (Noraxon Inc., Scottsdale, AZ, USA). Raw EMG signals were amplified (×1000), band pass filtered (10–500 Hz ± 2% cut-off) and full-wave rectified. Then, data were smoothed using root-mean-square analysis to evaluate the motor unit recruitment, which was calculated for a 100-ms window. During each of the 10-s cycling bout, the burst onset and offset of the rectified EMG profiles were defined as the signal that exceeded a threshold of 20% of the visually determined maximal EMG activity (da Silva et al., 2016).

#### HR Assessment

The HR was measured using a Garmin Forerunner 935 Fitness Watch monitor (Garmin International, Inc., Kansas, USA), and it was taken immediately following the cessation of each 10-s sprint cycling phase, to represent the physiological load of each sprint-cycling bout.

#### RPE

Immediately after each bout, participants provided their RPE value for the entire 10 s using the Borg CR-10 scale. On this scale, 0 point indicates ‘extremely easy’ and 10 points indicate ‘extremely difficult’.

### Statistical Analysis

Data were expressed as the mean ± SD. A two-way repeated-measures analysis of variance was used to quantify the differences in peak power, mean power, RMS EMG values, arousal score, HR, and RPE values. Significant condition × bout interactions were followed up with simple main-effects analyses. When no significant interaction was observed, the least significant difference (LSD) post hoc tests were used to follow up the significant main effects for each condition and set. Cohen’s *d* was used as an ES measurement for the comparison between two means and interpreted as small (0.2), medium (0.5), large (0.8) and very large (1.30) (Rosenthal, 1996). Statistical significance was set at *p* < 0.05. Statistical analyses were performed using IBM SPSS Statistics for Windows, version 25.0 (IBM Corp., Armonk, NY, USA).

## Results

**Figure 2:**
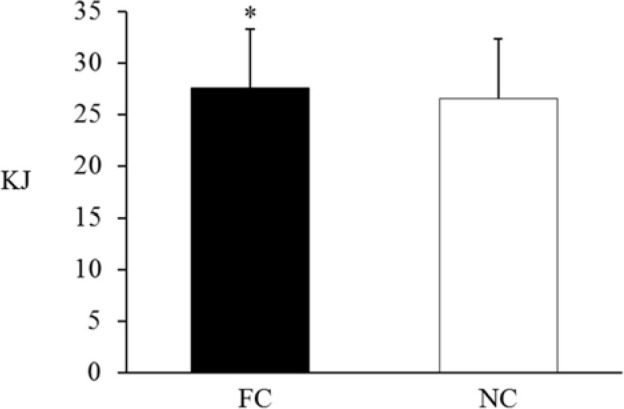
Total exercise volume of four bouts of 10-s sprint-cycling under the FC and NC conditions. * Significant difference between the FC and NC conditions. FC = foot cooling; NC = non-cooling.

Overall, as shown in Figure 2, the total work performed under FC was significantly higher than that under NC (27.57 ± 5.66 kJ vs. 26.55 ± 5.76 kJ; *t* = 4.783, *p* = 0.001, 95% CI = [0.54– 1.52] kJ, ES = 0.718).

A significant condition × bout interaction (*F* = 4.027, *p* = 0.017, ES = 0.309) was observed on mean power. The simple main effects of the condition were found on the third (*F* = 18.906, *p* = 0.002, 95% CI = [0.37–1.18] W/kg, ES = 0.677) and fourth bouts (*F* = 9.964, *p* = 0.012, 95% CI = [0.16– 0.96] W/kg, ES = 0.525). In comparison, a simple main effect of the bout was only detected under the NC condition (*F* = 3.590, *p* = 0.026, ES = 0.285). Pairwise comparisons revealed that under the NC condition, mean power was greater in the second than in the third and fourth bouts (*p* = 0.001 and 0.040, respectively), as presented in Table 1.

**Table 1:** Power output, FAS prior to each bout and EMG data from workouts under the FC and NC conditions (mean ± SD).

Variables	Bout 1	Bout 2	Bout 3	Bout 4
Mean power (W/kg)				
FC	9.98 ± 1.12	10.06 ± 1.19	10.14 ± 1.15*	9.79 ± 1.22*
NC	9.88 ± 1.38	9.95 ± 1.24	9.37 ± 1.30‡	9.23 ± 1.27‡
Peak power (W/kg)				
FC	11.88 ± 1.86	11.90 ± 1.17	12.09 ± 1.56	12.12 ± 1.50
NC	11.98 ± 2.00	12.14 ± 1.87	11.91 ± 1.54	11.69 ± 1.33
FAS				
FC	3.3 ± 1.4	4.3 ± 1.3*†	4.7 ± 0.9*†	5.1 ± 0.7*#‡†
NC	3.2 ± 0.9	3.6 ± 0.8	3.9 ± 0.9†	4.2 ± 1.1†
VL EMG (μV)				
FC	249.7 ± 57.6	254.1 ± 52.9	289.3 ± 66.7*†‡	296.0 ± 70.6*†‡
NC	255.6 ± 55.5	249.5 ± 47.1	263.6 ± 54.3	257.7 ± 54.5

* Significant difference between the FC and NC conditions. ^†^ Significant difference compared to the first bout. ^‡^ Significant difference compared to the second bout. ^#^ Significant difference compared to the third bout. FAS = felt arousal scale; EMG = electromyography; FC = foot cooling; NC = non-cooling; VL = vastus lateralis.

No significant condition (*F* = 0.061, *p* = 0.81, ES = 0.007), bout (*F* = 0.092, *p* = 0.964, ES = 0.01), or condition × bout interaction (*F* = 1.087, *p* = 0.372, ES = 0.108) effects were observed for peak power (Table 1).

A significant condition × bout interaction (*F* = 3.066, *p*= 0.045, ES= 0.254) was observed on the arousal score. The simple main effects of the condition on the second (*F* = 5.444, *p* = 0.045, 95% CI = 0.2–1.4, ES = 0.377), third (*F* = 7.579, *p* = 0.022, 95% CI = 0.1–1.5, ES = 0.457) and fourth (*F* = 5.651, *p* = 0.041, 95% CI = 0.4–1.8, ES = 0.386) bouts were identified. We also observed the simple main effects of bouts both under the FC (*F* = 9.705, *p* < 0.001, ES = 0.519) and NC (*F* = 4.796, *p* = 0.008, 0.348) conditions. Pairwise comparisons revealed that under the FC condition, the arousal level increased in the latter bouts (fourth > third = second > first), whereas under the NC condition, the arousal levels in the third and fourth bouts were significantly higher (*p* = 0.045 and 0.042, respectively) than that in the first bout, as indicated in Table 1.

We observed a significant condition × bout interaction (*F* = 4.878, *p* = 0.008, ES = 0.352). In addition, we identified the simple main effects of the condition on the third (*F* = 6.734, *p* = 0.029, 95% CI = [3.3–48.1] μV, ES = 0.428) and fourth (*F* = 10.253, *p* = 0.011, 95% CI = [11.2–65.4] μV, ES = 0.533) bouts. We observed a simple main effect of the bout, but only under the FC condition (*F* = 6.966, *p* = 0.001, ES = 0.436). Moreover, pairwise comparisons revealed that the RMS EMG values of the VL in the third and fourth bouts were significantly higher than those in the first (*p* = 0.002 and 0.018, respectively) and second (*p* = 0.018 and 0.039, respectively) bouts. The results are presented in Table 1.

Meanwhile, the RPE score and the HR are shown in Table 2. As presented, no condition × bout interaction (RPE score: *F*=1.121, *p*= 0.358, ES= 0.111; HR: *F*= 1.073, *p*= 0.377, ES = 0.107) or main effect of condition (RPE score: *F*= 0.126, *p*= 0.730, ES= 0.014; HR: *F* = 0.145, *p*= 0.712, ES= 0.016) was observed on the RPE values. Instead, only a significant main effect of the bout was found (RPE: *F* = 24.912, *p* < 0.001, ES= 0.735; HR: *F*= 24.275, *p*< 0.001, ES= 0.107). The LSD post hoc comparison showed that RPE scores differed among all bouts; furthermore, HR values in the second, third, and fourth bouts were significantly higher than in the first bout.

**Table 2:** HR and RPE during each bout of sprint cycling under the FC and NC conditions (mean ± SD).

Variables	Bout 1	Bout 2	Bout 3	Bout 4
RPE				
FC	5.4 ± 1.9	6.3 ± 1.8^†^	6.7 ± 1.8‡†	7.6 ± 1.8^#‡†^
NC	4.9 ± 1.8	5.8 ± 1.4^†^	6.8 ± 1.4‡†	7.7 ± 1.9^#‡†^
HR (beats/min)				
FC	153.2 ± 11.5	160.6 ± 7.35^†^	163.7 ± 7.5†	162.1 ± 8.4^†^
NC	150.8 ± 11.8	161.3 ± 10.5^†^	159.6 ± 7.6†	163.8 ± 7.5^†^

† Significant difference compared to the first bout. ^‡^ Significant difference compared to the second bout. ^#^ Significant difference compared to the third bout. HR = heart rate; RPE = rating of perceived exertion; FC = foot cooling; NC = non-cooling.

## Discussion

The primary findings of this study were mostly in favour of our research hypothesis, indicating that the arousal level, total work, mean power, and RMS EMG activities of the VL during the repeated sprint-cycling workout were higher under the FC than the NC condition. However, no differences were found in terms of peak power, the RPE, and the HR of the FC and NC conditions.

Previous studies have demonstrated that interset FC has an ergogenic effect on lower limb strength performance (Cai et al., 2021; Wu et al., 2021). The present study further demonstrated that in moderate ambient temperatures, FC enhanced repeated lower limb power performance (sprint cycling), displaying higher total work (Figure 2) and mean power by maintaining constant power output (Table 1) relative to NC; however, these results were not observed in all bouts overall. Our results are also roughly in accordance with those of studies on peripheral cooling of the palm conducted by Caruso et al. (2015), who reported that interset palm cooling had an ergogenic effect on the repeated submaximal level of concentric flywheel leg-press exercise by delaying average power decrements. However, the results of our study are in contrast with those of Caminita et al. (2020), who demonstrated that squat jump height and vertical ground-reaction force decreased after a 15-min cooling period using an ice-water bath (approximately 0°C) for the foot soles. Methodological differences, including cooling duration (2.5 min vs. 15 min) and cooling temperature (10°C vs. 0°C), may account for the discrepancy in findings because cooling reduces cutaneous sensory feedback (0°C, >10 min), which has detrimental effects on force production (Lowrey et al., 2013). Obviously, unlike previous cooling strategies on muscles used before (Cheung and Robinson, 2004) or between (Kim and Hurr, 2020) repeated sprint-cycling workouts that did not demonstrate ergogenic effects, the intermittent application of peripheral cooling on the foot took advantage of the cooling benefits on repeated power performance. However, although a previous study demonstrated that FC increased maximal lower limb muscular force (Wu et al., 2021), contrary to expectations, peak power during cycling (i.e., ability to exert force quickly) was not affected by FC. Our findings confirm previous evidence that only mean power and not peak power is affected by various intervention strategies during repeated sprint performance and dynamic resistance exercise workouts (Frikha et al., 2020; Wilk et al., 2020). Taken together, it appears that the peak power output, i.e., the task that requires an individual to exert maximal effort as rapidly as possible, may not be easily increased by interbout FC and other acute intervention strategies.

The arousal level estimated by FAS increased as the bout progressed, regardless of the condition. Moreover, the effect of FC magnified arousal levels during sprint-cycling workouts (Table 1). An elevated arousal level plays an ergogenic role in strength performance, such as producing more repetitions per set during a high-intensity resistance exercise workout (Wilk et al., 2020) and improving maximal motor strength over a short period (Perkins et al., 2001). Furthermore, during the 1 RM lower limb test, a higher arousal level elicited by FC was accompanied by increased 1 RM leg-press strength (Wu et al., 2021). The present findings suggest that an increase in the arousal level by interset FC may partly benefit the repeated power performance. However, it may be insufficient to explain the ergogenic effects because an FC-induced increase in the arousal level did not completely coincide with the increase in mean power, and FC did not elicit improvement in peak power during the sprint-cycling workout. Thus, additional factors rather than the arousal level alone are more likely the cause of higher mean repeated power performance obtained by interbout FC.

Our results also revealed that participants who were physically active showed decreased mean power output after the second bout under NC, suggesting that the repeated sprint-cycling protocol led to exercise fatigue (i.e., participants could not maintain constant power). However, regarding the RMS EMG activities of the VL under the NC condition, the agonist muscle activation remained unchanged after fatigue occurrence during repeated sprint cycling. In contrast, under the FC condition, participants exhibiting a steady level of mean power output which progressed through the given trial along with the increased RMS EMG activities of the VL in the last two bouts. Collectively, both the FC and NC conditions increased the EMG/power output ratio in the last two bouts, even if the FC condition had higher mean power and RMS EMG activities of the VL in the last two bouts than the NC condition (Table 1). The increased EMG/force ratio commonly accompanies peripheral fatigue and is often compensated by an increased EMG response for the force deficiency (Hug and Dorel, 2009). Accordingly, FC may delay peripheral fatigue by recruiting additional motor units from the agonist muscles to maintain a constant power output. The phenomenon wherein peripheral cooling increases corresponding muscle activities has been confirmed in previous studies in both active maximal voluntary contraction (Pietrosimone and Ingersoll, 2009) and passive electric stimulation (Hopkins et al., 2002; Palmieri-Smith et al., 2007). In addition, given that interset peripheral cooling was reported to accelerate metabolite clearance and increase subsequent power output (Caruso et al., 2015), the reason for FC facilitating recovery and reducing fatigue accumulated from the previous bouts which contributes to delaying the onset of fatigue during the repeated power exercise cannot be ruled out.

In the present study, significantly higher mean power in the last two bouts (Table 1) and total work volume (2.8%) were found between conditions that did not have any differences in HR and RPE values (Table 2). Our results reveal that participants performed more work with FC at a similar level of exertion. In accordance with our data, other researchers have reported that interset FC increased the total leg-press exercise volume, whereas there were no substantial physiological demands compared with NC (Cai et al., 2021). Previous studies have indicated that cooling induces temporary analgesia (Koc et al., 2006) and reduces pain perception (Verducci, 2000, 2001). Although these variables were not obtained in our study, they offer insights into the interpretation of mediating sensations of fatigue, which increases an individual’s tolerance during exercise and allows more work to be performed (Verducci, 2000, 2001). These results, along with those mentioned above, i.e., greater mean power, but no alternation in peak power under the FC condition during 10-s sprint cycling, suggest that during repeated power exercise, the ergogenic effect of interbout FC is to maintain constant power output and avoid a drastic reduction in power output either within or between bouts by increasing the central drive and recruiting more motor units of the main muscles to override fatigue and offset a power drop.

A limitation of the study is the power type exercise of cycling which involves mechanically low impact loading. Thus, the findings of our study might not be exactly appropriate to the performance of stretch-shortening cycle exercises, such as repeated jumps or field sprints. Despite the limitation, this study provides an ergogenic strategy for developing explosive power performance.

## Conclusions

The results of this study indicate that interbout FC (10°C, 2.5 min) elicits higher arousal levels and repeated lower limb power performance among physically active individuals. The ergogenic effect of interbout FC on repeated lower limb power exercise may delay peripheral fatigue by increasing the central drive and recruiting more motor units of the agonist muscles to compensate for fatigue-related responses and power decrements. Coaches and power athletes can use intermittent FC as part of their routine power training to enhance training responses and optimize lower limb power performance of athletes.
